# Floating Kidney; Its Symptoms and Surgery

**Published:** 1899-03

**Authors:** W. W. Hitchcock


### FLOATING KIDNEY; ITS SYMPTOMS AND SURGERY.

By W. W. HITCHCOCK, M.D.

My apology for bringing this subject before you for discussion is the almost total lack of reference to it in general medical literature and the varied neurotic and neurasthenic habit or temperament usually associated with a freely movable or floating kidney and the profound influences such a displaced kidney has, either mechanically or through reflex nervous disturbances, on the digestive and other organs. Certainly the two cases reported by me and subjoined to this paper for your thought and consideration present two very different and distinct groups of conditions and symptoms; one group consisting of pain and tenderness with dragging sensations and a sense of weight and fullness in the hypochondrium, all of which symptoms are directly referable to the kidney itself; and a second group entirely different from these, made up of loss of flesh and strength, palpitation, various gastric and intestinal disturbances, ovarian and uterine neuralgias, dysmenorrhea, accompanied by a condition of mental depression amounting at times to that of temporary insanity, and other evidences of apparent neurasthenia.

Whether this second group of symptoms is at all times a result of movable kidney, I am unable to say.

I am quite sure, however, that it is more than a coincidence.

Of the thirty-one cases collected and reported by Drummond, twenty-two were exceedingly nervous and might very properly be described as neurotics, and it is noteworthy that these were the cases in which the dyspeptic symptoms were most marked. The pronounced nervous symptoms so frequently observed, or what may be termed the neurotic habit, where there is a movable or floating kidney are so pronounced that we may well pause to inquire whether the symptoms enumerated are due to natural neurotic tendency of the patient or directly the result of the displaced organ. Some light may be suggested on this point by the knowledge we have acquired in the last few years from our surgical experience of the abdominal viscera in general and the digestive organs in particular.

We have learned that any movable organ situated in the abdominal cavity which becomes fixed by some of the many inflammatory conditions frequently resulting in adhesive bands due to exudates, set up many and varied distressing painful reflexes, and while this may be greatly exaggerated in those who are naturally susceptible through neurotic habit, yet it is impossible to entirely eliminate the mechanical element as playing an important influence as cause.

I call to mind a case bearing on this particular point, that of a man 42 years old who had received an injury of the abdomen by a kick of a horse from which he subsequently suffered an almost constant gnawing, dragging pain. On opening the abdomen a small band not more than a quarter of an inch wide had united one of the knuckles of the intestine to that of another; in fact, outside of the great annoyance experienced in finding the trouble, when found, so small and insignificant did it appear that no hope was felt that the liberation on the breaking-up of so small adhesion would relieve the patient, yet a cure was the result. I have had occasion to observe two similar cases in the practice of my colleagues where apparent trivial fixation of naturally movable organs produced most annoying paroxysmal distress accompanied by more or less constant dragging pain.

The question then naturally presents itself, would these same symptoms arise were it not for the natural neurotic tendency in the individual, or is the displaced organ the cause of the nervous condition? That in case of movable kidney the kidney condition determines the region and distribution of the disturbance, there can be no doubt, and further as in the condition cited above where there was no predisposition nor tendency to neurasthenia until after the inflicted injury had incited inflammatory processes, resulting in mechanical disturbances by adhesions—it will seem rational to suppose that in the majority of cases it is the cause of the neurasthenic conditions.

These functional neuroses accompanying movable kidney seem to be almost identical with those so commonly observed in cases of ovarian neuralgia where the ovaries, as far as we are able to determine, are in a healthy condition. Surgically these conditions are of the utmost importance in determining the prognosis and treatment, for if we are able to eliminate previous neuroses and fully decide in our mind that they are the result of mechanical disturbances and directly due to displaced kidney, nephrorraphy offers very positive assurance of success. On the other hand, when from the history we are unable to decide positively that the distress, pain : nd emaciation are not the result of this mechanical derangement, operative interference, like that of removal of healthy ovaries for pain, will result unsatisfactorily and in failure, though the operation so far as restoring the kidney to its normal anatomical position by various methods may succeed.

Mrs. C., aged 38, operated October 16th, 1884, until within the last eighteen months had alwas enjoyed good health; has been married fourteen years, borne three children, labors not difficult. For the las ten or twelve months had complained of pain in the left side of abdomen and in the region of the umbilicus, severe enough to prevent her from doing any but the very lightest work about the house. This pain had increased steadily, and what was especially noteworthy, was that pain was most severe when in the recumbent position and frequently of a colicky nature. She was a stout, healthy-looking woman, who evidently had been accustomed to hard work and possessed more than the ordinary amount of endurance.

Examination over the left side revealed an oval tumor, hard and freely movable, about the size of the closed fist. It could be moved down on a level with the umbilicus, and when held in this position there was an impulse transmitted to it from the abdominal aorta that suggested possible aneurism had it not been that pushing it out and upwards it could be made to disappear under the ribs. Firm pressure occasioned a peculiar sickening sensation of faintness and nausea not unlike that of pressing the ovary or testicle. The urine was normal. This case was apparently one of those belonging to the first class of individuals where the whole disturbance was occasioned by the mechanical condition alone. She had never manifested many of the peculiar nervous symptoms often found in these cases although there had been a loss of thirty pounds in weight. I advised nephorrhaphy which she submitted to with the hope of relief. An incision three and one-half inches in length extending from the twelfth rib downward and forward along the outer border of the right rectus muscle. The patient being of robust physique considerable difficulty was encountered in retracting the wound to enable the kidney to be brought into view. In order to make more room a counter incision was made at right angles forward about the middle of the first incision, which enabled me at once to recognize the fatty peri-renal capsule forming the bed of the kidney.

I further experienced more difficulty in finding an assistant with sufficient power and endurance to hold the kidney through the thick abdominal wall high enough and long enough in position after the fat had been cleared away, so that the kidney itself could be observed. The patient did not tolerate the ether kindly and some time was consumed in righting matters when, much to my surprise, I soon found the kidney was virtually coming into the wound and the assistant instead of pushing up against the abdominal wall until he was well nigh exhausted, now found it necessary to support the wound in order to prevent its escape. (Here was a lesson to me which I did not recognize for several years afterwards, viz.: that in kidney operations where the patient is placed in proper position, the kidney will come into its natural bed in the loin by simply the pressure of the abdominal viscera and the respiratory movements.) The wound was closed with carbolized silk suture, two passing through from without to the fascia, peri-renal fat and into the capsule of the kidney and out of the same on the opposite side. These were so tied that when the other stitches were placed in position they were a part of the general closure of the wound, but knotted so that they could be recognized as the ones passing through the peri-renal fat and capsule. A rubber drainage-tube was left in between the two sutures holding the kidney proper extending as far in as the capsule. This was removed the third day and the wound allowed to heal. The wound sutures were removed the tenth day while the two kidney sutures were left in until the fifteenth day.

Her recovery was uneventful, and she was permitted to leave her bed at the end of the fourth week. The operation relieved her of pain and all the distressing symptoms. I saw this woman the last time six years after the fixation and she was in good health and had regained her former weight.

The question that has frequently occurred to me in this case is, whether the firm adhesion in suturing was due to the engagement of the capsule, or whether it would not have been just as well had I simply inclosed the peri-renal fatty capsule and closed the wound? From observations made by Dunning on sheep and calves where he had experimented on the movable nature of the kidney soon after they were killed and hung up, it would seem that the peri-renal fat was the only substantial retaining buttress of the organ. He concludes from his studies that it is totally unnecessary to include the renal capsule in the suture.

Reasoning from the fact that we are dealing with a ruptured capsule in floating kidney, and taking into consideration the difficult task of being positive that we have sufficiently drawn up the fatty capsule in such a manner so as to completely close the rent, I have always felt safer in the use of the combined stitch, after pulling the fatty capsule well out, resecting it and closing it in.

Mrs. W., aged fifty-seven, came to me complaining of great pain and distress in the region of the abdomen and to the right of the median line. She said she had been practically bed-ridden for the last two years. She had the appearance of a typical neurasthenic and suffered with all the nervous symptoms usually observed in these cases.. Her dyspeptic symptoms were pronounced, she could not eat, and from her long continued nausea was in an extremely emaciated condition, having lost in the neighborhood of forty pounds in the last three years. She was occasionally attacked by what she was pleased to term congestions, at which time she would suffer the most excruciating pain of a colicky nature. She often became hysterical and despondent and resorted to all kinds of quack nostrums and pathies with the hope of finding relief. At the time she visited me at my office she remarked that she had made up her mind that she was on a tour of visitation to doctors to see if some one would not tell her something different from that which she had so frequently heard, viz.: that she was an old chronic hysterical dyspeptic. Having already consumed the major part of my office hour I made an engagement to see her at her home the next day.

Examination over the right side revealed a distinct lump or tumor about the size of a small orange which was freely movable and could be pushed backwards and upwards into the renal pouch. Manipulation excited nausea and a sense of faintness, and there was a great tenderness over the whole surface of the abdomen.

She was troubled greatly with flatulence, constipation, with occasional attacks of diarrhea and general digestive disturbance, followed by nervous prostation confining her to bed for several days at a time.

Diagnosis, Floating Kidney—Fixation was advised, which she submitted to on September 9, 1898, at the California hospital. Sufficient time, has not elapsed to determine a permanent cure, but at this time, eight weeks after the operation, she is greatly relieved, gaining in weight and strength with fair hopes of perfect cure.

Conclusions and Deductions.—Normally the kidney is more or less movable, and what is ordinarily denominated movable kidney in contra-distinction to that of floating kidney is congenital, while floating kidney is always acquired. No one now believes that a movable kidney is a menace to its possessor's health, and should either be removed or immediately fixed. No kidney should be fixed unless its mobility occasions a series of symptoms that interferes sufficiently with one's ordinary occupation or by its mechanical irritation of other organs leads to emaciation, neurasthenia or distressing painful paroxysms. Nephrorrhaphy, where indicated, is conservative surgery in that it will prevent the necessity in many cases of subsequently doing a nephrectomy which may become necessary, first, by the bending of the ureter and giving rise to hydronephrosis which may become converted into pyonephrosis; secondly, by dragging on the abdominal aorta and kinking the vena-cava, a condition simulating aneurism may be produced.

Pain of a character referred to the region of distribution of the spinal nerves is often produced by a too movable kidney through disturbance of the abdominal plexus. A nerve exhaustion or neurasthenia may result by interference with digestion, assimilation and absorption.

All cases of too movable kidney if accompanied by symptoms pointing directly to the kidney as the source should be operated.

Nephrorrhapy under strict aseptic precautions is a safe and usually effective procedure. The correctness of these deductions have frequently been demonstrated by the disappearance of all the symptoms after a restoration and retention of the kidney in its normal position.—*Southern California Practitioner.*

